# Anti-TNF-Alpha-Adalimumab Therapy Is Associated with Persistent Improvement of Endothelial Function without Progression of Carotid Intima-Media Wall Thickness in Patients with Rheumatoid Arthritis Refractory to Conventional Therapy

**DOI:** 10.1155/2012/674265

**Published:** 2012-07-31

**Authors:** Carlos Gonzalez-Juanatey, Tomas R. Vazquez-Rodriguez, Jose A. Miranda-Filloy, Ines Gomez-Acebo, Ana Testa, Carlos Garcia-Porrua, Amalia Sanchez-Andrade, Javier Llorca, Miguel A. González-Gay

**Affiliations:** ^1^Cardiology Division, Hospital Universitario Lucus Augusti, c/Ulises Romero 1, 27003 Lugo, Spain; ^2^Rheumatology Division, Hospital Universitario Lucus Augusti, c/Ulises Romero 1, 27003 Lugo, Spain; ^3^Epidemiology and Computational Biology Division, School of Medicine, University of Cantabria, Avenida Herrera Oria s/n, 39011 Santander, Spain; ^4^Rheumatology Division, Hospital Universitario Marques de Valdecilla, Avenida de Valdecilla s/n, 39008 Santander, Spain

## Abstract

To determine whether treatment with the anti-TNF-alpha blocker adalimumab yields persistent improvement of endothelial function and prevents from morphological progression of subclinical atherosclerosis in patients with rheumatoid arthritis (RA) refractory to conventional therapy, a series of 34 consecutive RA patients, attending hospital outpatient clinics and who were switched from disease modifying antirheumatic drug therapy to anti-TNF-alpha-adalimumab treatment because of severe disease, were assessed by ultrasonography techniques before the onset of adalimumab therapy (at day 0) and then at day 14 and at month 12. Values of flow-mediated endothelium-dependent vasodilatation at day 14 and at month 12 were significantly higher (mean ± standard deviation (SD): 6.1 ± 3.9%; median: 5.7% at day 14, and mean ± SD: 7.4 ± 2.8%; median: 6.9% at month 12) than those obtained at day 0 (mean: 4.5 ± 4.0%; median: 3.6%; *P* = 0.03 and *P* < 0.001, resp.). Endothelium-independent vasodilatation results did not significantly change compared with those obtained at day 0. No significant differences were observed when carotid artery intima-media wall thickness values obtained at month 12 (mean ± SD: 0.69 ± 0.21 mm) were compared with those found at day 0 (0.65 ± 0.16 mm) (*P* = 0.3). In conclusion, anti-TNF-alpha-adalimumab therapy has beneficial effects on the development of the subclinical atherosclerosis disease in RA.

## 1. Introduction

Rheumatoid arthritis (RA) is a chronic inflammatory disease associated with accelerated atherosclerosis and increased incidence of cardiovascular (CV) events [1]. Besides a genetic component [2] and classic (traditional) CV risk factors [3], chronic inflammation plays a pivotal role in the development of atherogenesis in patients with RA [4]. Different validated techniques are currently available to determine subclinical atherosclerosis in patients with rheumatic diseases. Macrovascular endothelial dysfunction, an early stage in atherosclerosis, can be detected by brachial ultrasonography as the result of impaired flow-mediated endothelium-dependent vasodilatation (FMD). Carotid ultrasound studies are also useful to disclose the presence of subclinical atherosclerosis [5]. By this technique, morphological changes such as abnormally increased carotid artery intima-media wall thickness (IMT) and carotid plaques can be observed [5].

A number of studies have shown short-term improvement of endothelial function in RA refractory to disease modifying antirheumatic drugs (DMARDs) following anti-TNF-alpha therapy [6, 7]. However, carotid ultrasound studies in patients with RA undergoing anti-TNF-alpha therapy have yielded contradictory results in terms of reduction or progression of carotid IMT [8–10]. Nevertheless, from a clinical point of view, anti-TNF-alpha therapy has been associated with a decrease in the incidence of CV events in patients with RA. In this regard, results from the British Society for Rheumatology Biologics Register showed that the risk of myocardial infarction was markedly reduced in RA patients who responded to anti-TNF-alpha therapy by 6 months compared with nonresponders [11]. Also, in a study that included 10156 RA patients enrolled in the Consortium of Rheumatology Researchers of North America, individuals using a TNF-alpha antagonist experienced a reduced risk of the primary composite CV endpoint compared with users of nonbiological DMARDs [12]. In keeping with these observations, data from a recent systematic review confirmed that anti-TNF-alpha therapy was associated with a reduced risk for all CV events, myocardial infarction, and cerebrovascular accidents [13]. Meta-analysis of randomized controlled trials also yielded a point estimate indicating a lower risk of CV events in patients undergoing anti-TNF-alpha therapy [13].

Taking these observations together, in an attempt to further investigate the potential beneficial effect of TNF-alpha antagonist therapy on subclinical atherosclerosis in RA, we sought to determine whether adalimumab therapy might yield persistent improvement of endothelial function and no morphological progression of subclinical atherosclerosis measured by the determination of carotid artery IMT in RA patients with severe disease, refractory to DMARDs, who were prospectively followed over 1 year period.

## 2. Materials and Methods

### 2.1. Patients

A series of consecutive RA patients that fulfilled the 1987 American College classification criteria for RA [14], attending hospital outpatient clinics from Hospital Xeral-Calde (Lugo, NW Spain), who were switched from standard DMARD therapy to anti-TNF-alpha-adalimumab treatment between April 2008 and May 2009 because of severe and active disease (DAS28 greater than 5.1) [15], were assessed before the onset of adalimumab therapy and then prospectively until 1 year after the commencement of treatment with this therapy.

For the purpose of this study, RA patients seen during the period of recruitment with diabetes mellitus, current smokers, history of coronary heart disease, heart failure, stroke, peripheral arteriopathy, estimated pulmonary artery systolic pressure greater than 35 mmHg, mitral, aortic, tricuspid, pulmonary valve involvement (regurgitation or stenosis), pericardial effusion in an echocardiography study performed at the time of recruitment, or body mass index less than 20 or greater than 35 Kg/m^2^ were excluded.

Based on the inclusion and exclusion criteria, we recruited 34 RA white patients (30 women, 28 (82.4%) of them rheumatoid factor positive). The median age at the time of disease diagnosis was 50.1 (interquartile range (IQ) 41.3–55.9) years. The delay to the diagnosis of RA from the onset of symptoms was 0.5 (IQ range 0.3–1.6) years. The age at the onset of adalimumab therapy was 54.9 (IQ range 47.5–63.0) years. At the commencement of adalimumab, 26 patients were on methotrexate (MTX) therapy (median 15 mg/week) and 14 on leflunomide (20 mg/day), some of them receiving combination therapy with these two DMARDs. Six of the 34 patients were also receiving hydroxychloroquine (median 200 mg/day). Twenty-four patients were on prednisone (median 5 mg/day). Five received nonsteroidal anti-inflammatory drugs. Nine patients had a history of hypertension. However, in each case appropriate control of blood pressure was achieved following treatment with antihypertensive drugs. Seven patients received statins because of hypercholesterolemia. Another 7 were ex-smokers.

None had ever used nitrates or were on treatment with estrogens.

Adalimumab therapy (40 mg) was subcutaneously administered every 2 weeks over the period of study. Concomitant medication was not changed during the period of study.

The Galician ethical Committee approved this study. Also, patients signed and informed consent to participate in this study.

### 2.2. Study Protocol

Patients received adalimumab therapy 40 mg every other week by subcutaneous injection from day 0 (onset of study) to month 12 (end of the study) 1 year after the initiation of adalimumab therapy.

In each patient, a DAS 28 (0–10) (determined in each patient by the same rheumatologist throughout the study) using erythrocyte sedimentation rate (ESR) as the laboratory datum was assessed at day 0 (two hours before the first administration of adalimumab), at day 14 (two hours before the second administration of adalimumab), and at month 12 (two hours prior to adalimumab administration). Systolic and diastolic blood pressure, C-reactive protein (CRP-immunoturbidity method), ESR (Westergren), and serum creatinine were also determined at day 0 (two hours before the first administration of adalimumab), at day 14 (two hours before the second administration of adalimumab), and at month 12 (two hours before adalimumab administration). Furthermore, total cholesterol, triglycerides, LDL cholesterol, HDL cholesterol (fasting overnight determinations), and total cholesterol/HDL ratio (atherogenic index) were assessed at day 0, and at month 12.

Endothelial function was assessed before the first administration of adalimumab (two hours before) at day 0 at day 14 (two hours before the second administration of adalimumab), and at month 12 (at the end of the study, two hours before adalimumab administration).

Carotid artery IMT was measured before the first administration of adalimumab (two hours before) and at month 12 (at the end of the study).

Flow-mediated endothelium-dependent vasodilatation (postischemia) FMD and independent vasodilatation (postnitroglycerin) NTG were measured by brachial ultrasonography. Brachial artery diameter and flow were determined as previously described [16, 17]. B-mode scan of the right brachial artery, in a longitudinal Section 2 to 12 cm proximal to the antecubital fossa, was performed in supine subjects using a Philips IE33 (Philips Healthcare, DA Best, The Netherlands) with a 11 MHz linear transducer. The anterior and posterior media-intima interfaces were used to define the baseline artery diameter, calculated as the average of measurements made during four cardiac cycles at end diastole. The forearm blood pressure cuff was inflated on the ipsilateral wrist to 50 mmHg above resting systolic blood pressure for 5 minutes and then released. FMD (an increase in brachial artery diameter) was measured from 30 to 60 seconds after cuff release. To assess NTG endothelium-independent vasodilatation, we used 400 *μ*g of sublingual nitroglycerin, which acts directly on vessel smooth muscle to cause vasodilatation. NTG was measured 4 minutes after nitroglycerin intake. Intraobserver variability showed the following coefficients of variation: FMD 1.3% and NTG 1.9%.

Assessment of carotid artery IMT was performed as previously described in recent studies from our group [18, 19]. Briefly, carotid IMT was measured in the right common carotid. The study was performed using high-resolution B-mode ultrasound (Philips IE33; Philips Healthcare, DA Best, The Netherlands) with an 11 MHz linear transducer. For the purpose of the present study, QLAB's IMT-quantification software measurement plug in (Philips Healthcare, DA Best, The Netherlands) was used to increase the consistency and reliability of IMT measurements, reduce the effort required to successfully carry out IMT measurements, and minimize the time needed to complete an IMT study. The reproducibility of the IMT measurements was evaluated in 10 patients within 1 week of the first ultrasound examination. The correlation coefficient for carotid IMT was 0.985.

In all cases a cardiologist (CG-J) analyzed ultrasound data offline and he was blind to the clinical information and study date.

### 2.3. Statistical Analysis

Data were expressed as mean ± SD, median, and IQR. Measurements of FMD and NTG represented the maximal increase in brachial diastolic artery diameter and were expressed as percentage of change (%) from baseline. Equality of values at day 0 versus day 14 and at day 0 versus month 12 was tested using the Wilcoxon matched-pairs signed-rank test. All tests were two tailed. Statistical significance was accepted at *P* < 0.05.

Figures on the evolution from day 0 on (onwards) were obtained via locally weighted regression; its results were displayed with three curves representing the central estimate and lower and upper limits for confidence bands.

## 3. Results

**Figure 1:**
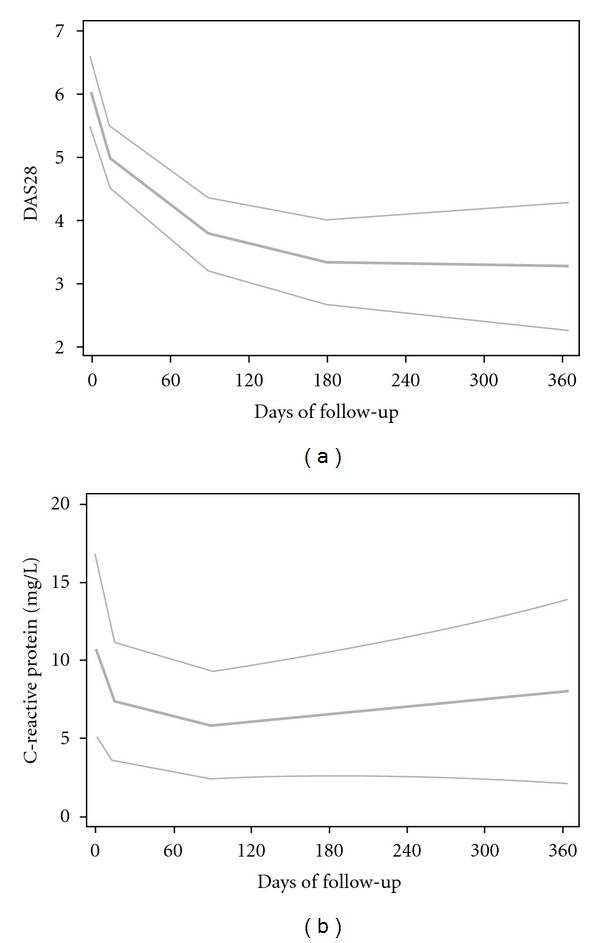
Changes in DAS28 (a) and C-reactive protein (b) from day 0 onwards, obtained via locally weighted regression. Central estimate and lower and upper limits of 95% confidence bands.

The use of anti-TNF-alpha-monoclonal antibody-adalimumab yielded clinical improvement in this series of RA with severe disease refractory-to-conventional DMARD therapy (Figure 1). DAS28 values at month 12 were significantly reduced (mean ± SD: 3.3 ± 1.5; median: 3.3) when compared to those observed before the onset of adalimumab therapy at day 0 (mean ± SD: 5.9 ± 0.7; median: 5.9; *P* < 0.001) (Table 1). Moreover, a significant reduction in the serum levels of CRP was achieved following the administration of adalimumab at day 14 (median: 4.9 mg/L) compared to baseline levels observed at day 0 (median: 9.1 mg/L; *P* = 0.008) (Table 1).

However, no statistically significant differences were found when total cholesterol, HDL-cholesterol and LDL-cholesterol, levels observed at month 12 were compared with those found at day 0. This was also the case for the atherogenic index (Table 2).

**Figure 2:**
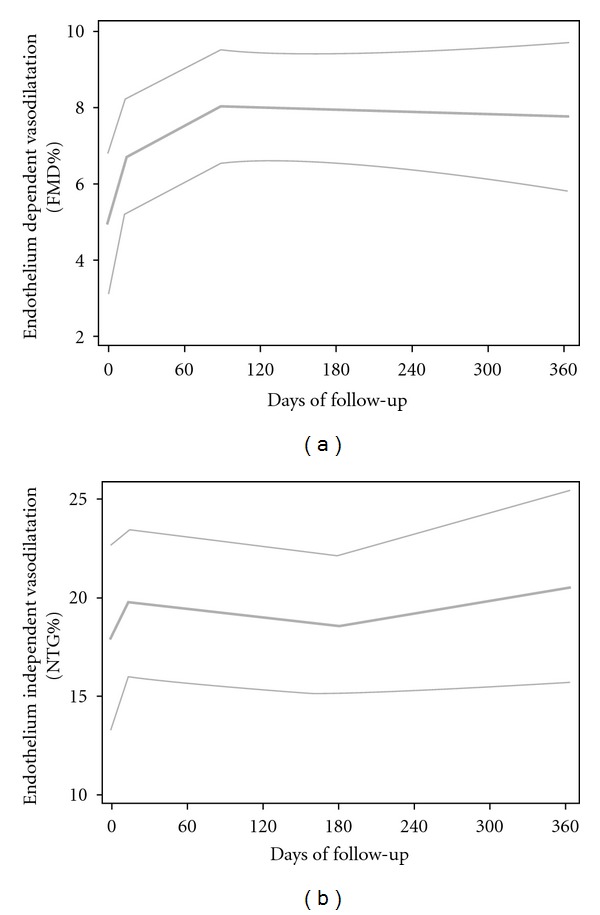
Changes in flow-mediated endothelium-dependent (FMD%) (a) and endothelium-independent (NTG%) vasodilatation (b) from day 0 onwards, obtained via locally weighted regression. Curves are central estimate and lower and upper limits of 95% confidence bands.

As previously described in RA patients with long-standing disease [20], this series of RA patients also had endothelial dysfunction prior to the onset of adalimumab therapy as the mean and median FMD percentage values were lower than 7% [21]. In addition, the present study confirmed a short-term rapid and significant improvement of endothelial function following the first administration of adalimumab [7]. In this regard, at day 14, values of FMD percentage were significantly higher (mean ± SD: 6.1 ± 3.9%; median: 5.7%) than those observed at day 0 (mean ± SD: 4.5 ± 4.0%; median: 3.6%; *P* = 0.03) (Table 1). Moreover, persistent improvement of endothelial function was observed at the end of the study (Figure 2). With respect to this, values of FMD percentage at month 12 (mean ± SD: 7.4 ± 2.8%; median: 6.9%) were significantly higher than those observed at day 0 (*P* < 0.001) (Table 1). However, no statistically significant differences were achieved when NTG percentage values observed at day 0 were compared with those obtained at day 14 or at month 12 (Table 1 and Figure 2).

No statistically significant changes were found when carotid artery IMT results obtained at month 12 (mean ± SD: 0.69 ± 0.21 mm; median: 0.68) were compared with those obtained at day 0 (mean ± SD: 0.65 ± 0.16 mm; median: 0.64; *P* = 0.30) (Table 1). Therefore, no significant morphological progression of subclinical atherosclerosis was observed in this series of adalimumab-treated RA patients.

## 4. Discussion

The present study shows persistent improvement of endothelial function following the TNF-alpha antagonist adalimumab in a cohort of patients with RA refractory to conventional DMARD therapy. Also, unlike our previous observations on patients with severe disease undergoing treatment with the chimeric anti-TNF-alpha monoclonal antibody-infliximab [8], no progression of subclinical atherosclerosis was observed after 1 year of adalimumab therapy.

A recent study on 8 early RA patients (disease duration ≤ 1 year), treated with adalimumab during 6 months, showed significant improvement of endothelial function that inversely correlated with CRP levels [22]. Sidiropoulos et al. also demonstrated an improvement of endothelial function after 18 months therapy with infliximab or adalimumab [23]. In keeping with these observations, we observed that adalimumab therapy yielded improvement of endothelial function after 12 months of therapy. However, the beneficial effect on endothelial dysfunction does not seem to be specific of anti-TNF-alpha drugs as therapy with rituximab, a monoclonal antibody that selectively targets CD20 positive B cells, demonstrated in two different studies an early and sustained favorable effect on endothelial function in RA patients refractory to TNF-alpha blockers [17, 24]. In a former study of our group, we demonstrated that in long-term anti-TNF-*α* infliximab-treated RA patients, the intravenous infusion of this monoclonal antibody yielded a significant rapid but transient improvement of endothelial dysfunction [25]. In this regard, following infusion of the drug, a dramatic and rapid increase in the percentage of FMD was observed. In all patients, percentages of FMD were greater than those observed 2 days before infusion. However, of FMD percentage values returned to baseline by 4 weeks after infusion of the drug. Therefore, we think differences in the bioavailability of the different TNF-*α* blockers may be a possible explanation for the persistent positive effect of adalimumab on endothelial function in long-term RA patients when compared with data obtained in long-standing RA patients following a single infusion of infliximab.

A result derived from the present study is that the improvement of endothelial function in RA seems to be independent of the effect of these biologic agents on the lipid profile. In this regard, although dyslipidemia is also closely linked to the development of endothelial dysfunction and atherosclerosis [1], short-term infliximab therapy was associated with significant increase of both total cholesterol and HDL cholesterol levels [26], plasma total cholesterol concentrations, LDL cholesterol concentrations, and also the atherogenic index increased after 1 year from the start of this therapy [26]. In accordance with these observations, in our study we did not observe a significant change in the atherogenic index when results obtained at month 12 were compared with baseline results.

Atherosclerosis is increasingly considered to be an immune system-mediated process of the vascular system and the inflammatory process taking place in the arterial wall as part of atherosclerosis disease. The actual evidence suggests that proinflammatory cytokines and metabolic abnormalities associated with systemic inflammation may be implicated in the development of endothelial dysfunction in RA. The chronic inflammation may lead to endothelial dysfunction, subsequent atherosclerosis, and cardiovascular events in RA [1].

Regardless of the biologic agent used (viz., adalimumab, infliximab, or rituximab), improvement of endothelial dysfunction has been demonstrated in RA patients that exhibit low levels of inflammation and clinical remission. In this regard, RA patients with early diagnosed disease treatment with adalimumab yielded improvement of endothelial function that was significantly related to clinical remission [22]. In keeping with that, although in our study reduction of blood pressure following 12-month adalimumab therapy did not reach statistical significance, it is remarkable to see that both the mean systolic and diastolic blood pressure were reduced in adalimumab-treated patients when compared with baseline results of blood pressure obtained at the onset of this biologic therapy. To the best of our knowledge, this reduction of blood pressure levels in long-term adalimumab-treated patients has not previously been reported. Whether the reduction of blood pressure levels following 12-month adalimumab therapy is the consequence of the decrease of the systemic inflammatory burden is a question that still remains unanswered. However, we feel that it may be a plausible explanation for these findings.

Carotid IMT is a useful surrogate marker of subclinical atherosclerosis and a predictor of CV events in patients with RA [27]. Due to this, as discussed before, another important result derived from the present study was the lack of significant increase of the carotid IMT following 12 months of adalimumab therapy. Previous studies on high resolution B-mode ultrasound of the common carotid artery disclosed a strong correlation between the carotid IMT and markers of systemic inflammation in both controls and patients with RA [4, 28]. Studies that addressed the effect of the control of CV risk factors on the progression of atherosclerosis, as measured by carotid IMT, used the concept of “change in the progression of IMT” because progression in the measurements of carotid IMT is expected with time/age [29] and because a reduction in this progression should be of great clinical significance. With respect to this, anti-TNF blocking agents, but not MTX, have been found to reduce carotid IMT in patients with RA [9, 10, 22]. In line with these studies, no progression in the carotid IMT was found in our series of adalimumab-treated patients. However, despite having a reduction in markers of inflammation, we could not disclose a decrease in the carotid IMT in our series. Nevertheless, the absence of carotid IMT progression enhances the potential usefulness of the TNF-alpha blocker therapy to decrease the accelerated atherosclerosis observed in patients with RA.

## 5. Conclusion

Twelve-month adalimumab therapy reduces mechanisms implicated in the increased risk of CV death observed in patients with RA.

## Figures and Tables

**Table 1:** Changes in DAS28, CRP, and ultrasonography data in 34 patients undergoing anti-TNF-alpha-adalimumab therapy due to RA refractory to conventional DMARDs.

	Day 0				Day 14				Month 12				Day 0 verus Day 14	Day 0 versus Month 12
Variable	Mean	±SD	Median	(IQR)	Mean	±SD	Median	(IQR)	Mean	±SD	Median	(IQR)	*P*	*P*
FMD%	4.5	±4.0	3.6	(2.1–7.0)	6.1	±3.9	5.7	(2.9–8.7)	7.4	±2.8	6.9	(5.4–9.2)	0.03	<0.001
NTG%	19.3	±7.5	19.5	(14.8–24.2)	20.1	±8.9	19.6	(15.0–27.5)	22.3	±7.8	19.7	(16.5–24.6)	0.52	0.08
Carotid IMT (mm)	0.65	±0.16	0.64	(0.52–0.75)					0.69	±0.21	0.68	(0.53–0.79)		0.30
DAS28	5.9	±0.7	5.9	(5.4–6.4)	4.5	±1.1	4.6	(4.0–5.3)	3.3	±1.5	3.3	(2.1–4.2)	<0.001	<0.001
CRP (mg/L)	15.6	±16.6	9.1	(3.5–21.0)	8.9	±14.0	4.9	(1.2–8.5)	6.8	±11.8	3.0	(1.1–8.4)	0.008	0.07

(FMD: flow-mediated endothelium dependent vasodilatation; NTG: flow-mediated endothelium independent vasodilatation; IMT: intima-media thickness; CRP: C-reactive protein).

**Table 2:** Changes in the lipid profile and blood pressure levels in 34 patients undergoing anti-TNF-alpha-adalimumab therapy due RA refractory to conventional DMARDs.

Variable	Day 0		Month 12		Day 0 versus month 12
	Mean	±SD	Mean	±SD	*P*
Total cholesterol (mg/dL)	206.1	±33.5	208.2	±40.7	0.94
LDL-cholesterol (mg/dL)	125.4	±4.8	124.8	±38.8	0.90
HDL-cholesterol (mg/dL)	60.4	±15.8	61.8	±14.7	0.96
Atherogenic index	3.61	±1.03	3.51	±0.98	0.73
Triglycerides (mg/dL)	101.6	±5.5	108.7	±4.1	0.31
Systolic blood pressure (mmHg)	136.0	±17.8	126.9	±18.2	0.10
Diastolic blood pressure (mmHg)	81.6	±9.6	79.2	±11.6	0.36
